# IL1 Receptor Antagonist Gene *IL1-RN* Variable Number of Tandem Repeats Polymorphism and Cancer Risk: A Literature Review and Meta-Analysis

**DOI:** 10.1371/journal.pone.0046017

**Published:** 2012-09-25

**Authors:** Ying Zhang, Changming Liu, Huiping Peng, Jianzhi Zhang, Quanlin Feng

**Affiliations:** 1 Inspection Division, Kunshan Hospital Affiliated to Nanjing University of Chinese Medicine, Kunshan, Jiangsu, China; 2 Department of Gastroenterology, Kunshan Hospital Affiliated to Nanjing University of Chinese Medicine, Kunshan, Jiangsu, China; 3 Department of Surgical Oncology, Kunshan Hospital Affiliated to Nanjing University of Chinese Medicine, Kunshan, Jiangsu, China; National Cancer Center, Japan

## Abstract

IL1 receptor antagonist (IL1RA) and IL1beta (IL1β), members of the pro-inflammatory cytokine interleukin-1 (IL1) family, play a potential role against infection and in the pathogenesis of cancers. The variable number of tandem repeats (VNTR) polymorphism in the second intron of the IL1 receptor antagonist gene (*IL1-RN*) and a polymorphism in exon 5 of *IL1B* (*IL1B*+3954C>T, rs1143634) have been suggested in predisposition to cancer risk. However, studies have shown inconsistent results. To validate any association, a meta-analysis was performed with 14,854 cases and 19,337 controls from 71 published case–control studies for *IL1-RN* VNTR and 33 eligible studies contained 7,847 cases and 8917 controls for *IL1B* +3954. Odds ratios (ORs) with 95% confidence intervals (CIs) were calculated from comparisons to assess the strength of the association. There was significant association between the *IL1-RN* VNTR polymorphism and the risk of cancer for any overall comparison. Furthermore, cancer type stratification analysis revealed that there were significantly increased risks of gastric cancer, bladder cancer and other cancer groups. Infection status analysis indicated that the *H. pylori* or HBV/HCV infection and *IL1-RN* VNTR genotypes were independent factors for developing gastric or hepatocellular cancers. In addition, a borderline significant association was observed between *IL1B*+3954 polymorphism and the increased cancer risk. Although some modest bias could not be eliminated, this meta-analysis suggested that the *IL1-RN* VNTR polymorphisms may contribute to genetic susceptibility to gastric cancer. More studies are needed to further evaluate the role of the *IL1B*+3954 polymorphism in the etiology of cancer.

## Introduction

Interleukins (ILs) are pro-inflammatory cytokines produced by monocytes, macrophages and epithelial cells. The interleukin-1 (IL1) family consists of the cytokines IL1alpha (IL1α), IL1beta (IL1β) and a specific receptor antagonist (IL1RA) [1]. The genes encoding this family are mapped on chromosome 2q14 [2] and include three related genes, *IL1A*, *IL1B* and *IL1-RN*, which encode IL1α, IL1β and IL1RA respectively. *IL1B* may play an essential role in the carcinogenic process. Several *IL1B* gene single nucleotide polymorphisms have been described to be associated with cancers to date [3]. Previously, we have reported the polymorphism in the promoter of *IL1B*(*IL-1B* -31T>C, rs1143627) was a low-penetrance protective factor for the development of cancer [4]. In addition, a polymorphism in exon 5 of *IL1B*(*IL1B* +3954C>T, rs1143634) has been found to affect *IL1B* gene expression [5] and to be associated with occurrence of cancers [6], [7]. IL1RA competitively binds to IL1 receptors with IL1α and IL1β. Naturally occurring anti-inflammatory IL1RA is a 16 to 18 kD protein that competes with the binding of IL1 to its receptor. In the second intron of the *IL1-RN* gene, there exists a variable number of tandem repeats (VNTR) with an 86 base pair nucleotide sequence as its repeating element. This region contains three potential protein binding sites: an interferon α silencer A, an interferon β silencer B and an acute phase response element, and thus may be of functional relevance [8]. It has been reported that these three binding sites affect the control of cell proliferation activity, resulting in potential regulation of IL1RA production [5], [8]. El-Omar et al. [9] first reported positive association between gastric cancer risk and these VNTR polymorphisms in *IL1-RN*. Another study revealed that *IL1-RN* VNTR genotypes were highly correlated with cellular function. The IL-1RN alleles were coded as previously described: allele 1, four repeats; allele 2, two repeats; allele 3, five repeats; allele 4, three repeats and allele 5, six repeats. The *IL-1RN* alleles were further divided into two categories: long genotype (L: including alleles 1, 3, 4, and 5) and short genotype (2: allele 2 only). The genotypes were classified as LL, 2L, and 22 [10].

A large number of genetic susceptibility studies have been carried out to investigate the associations between the *IL1B*+3954 and *IL1-RN* polymorphism and cancers of different tissue origins, but inconsistent results have been obtained. These inconsistent findings might be caused by a range of factors such as heterogeneity by cancer subtype, limited sample size, and gene-environment interactions. To further assess and validate the association of the *IL1B* +3954 and *IL1-RN* VNTR polymorphism with cancer risk, a comprehensive review and analysis of published data from different study groups is urgently needed. In this study, we have extensively reviewed literature and performed a meta-analysis based on all eligible case-control published data to evaluate the association between the *IL1B*+3954 and *IL1-RN* VNTR polymorphism and cancer susceptibility.

## Materials and Methods

### Identification of Eligible Studies

We searched the PubMed and Embase databases for all relevant reports (updated to May 23, 2012) by using the key words ‘interleukin-1 receptor antagonist’, ‘IL1’, ‘polymorphism’, or ‘cancer’. The search was limited to English language papers and human subject studies. In addition, studies were identified by a manual search of the reference lists of reviews and retrieved studies. When more than one of the same or overlapping populations by different investigators or overlapping data by the same authors were found, only the most recent or complete study was used for this meta-analysis. Studies, regardless of sample size, were included if they met the following criteria: (i) the study was of the *IL1B*+3954 and *IL1-RN* VNTR polymorphism and cancer risk, (ii) it was a case–control designed study and (iii) it contained available genotype frequencies (Figure S1).

### Data Extraction

Two individuals (Ying Zhang and Changming Liu) extracted all data independently complying with the selection criteria. For each study, the following characteristics were extracted: the first author’s last name, year of publication, country of origin, ethnicity, matching numbers of genotyped cases and controls, the source of control groups (population- or hospital-based controls), genotyping methods and cancer type. Different ethnic descents were categorized as Caucasian, Asian or mixed, which included more than one ethnic descent. One study included the information for genotypes of LL/2L and 22, without data for LL and 2L genotypes, so we were only able to calculate the OR for the comparison between alleles 2 and L [11]. In addition, the infection status of cases and controls in the enrolled studies were also extracted for the *IL1-RN* VNTR subgroup analysis.

### Statistical Analysis

The *IL1B*+3954 and *IL1-RN* VNTR polymorphisms were tested for associations with cancer susceptibility based on different genetic models. The meta-analysis examined the overall association of the *IL1B*+3954 and *IL1-RN* VNTR polymorphism with the risk of cancer measured by odds ratios (ORs) with 95% confidence intervals (CIs). To contrast the wild-type homozygote (*IL1B*+3954-CC or *IL1-RN*-L/L), we first estimated the risks of the *IL1B*+3954 -CT, -TT and *IL1-RN* -2/L, -2/2 genotypes on cancers, then evaluated the risk of cancer under a dominant model(*IL1B*+3954:T/T+C/T versus CC; *IL1-RN*:2/2+2/L versus L/L). In addition, recessive model associations were also estimated (*IL1B*+3954: T/T versus C/C+C/T; *IL1-RN*: 2/2 versus L/L +2/L). Stratified analyses were also performed by ethnicity (Asian, Caucasian and Mix), cancer types (if only one cancer type contained fewer than three individual studies it was combined into the ‘Other Cancers’ group), moreover, subgroup analysis was carried by infection status for *IL1-RN* VNTR polymorphism.

The statistical significance of the pooled OR was determined with the Z test, a P value of<0.05 was considered significant. The heterogeneity between studies was evaluated by the chi-square based Q statistical test [12], heterogeneity was considered significant for P<0.05. A fixed-effect model using the Mantel–Haenszel method and a random-effects model using the DerSimonian and Laird method were used to pool the results [13]. The random-effects model was used when there was heterogeneity in the results of the studies; otherwise, the fixed-effect model was used. Sensitivity analyses were performed to assess the stability of the results, namely, a single study in the meta-analysis was deleted each time to reflect the influence of the individual data set to the pooled OR. To test for publication bias, Funnel plots and Egger’s linear regression test were applied [14].

All statistical tests for this meta-analysis were performed with STATA version 10.0 (Stata Corporation College Station, TX), and SAS (version 9.1; SAS Institute, Cary, NC).

## Results

### Characteristics of the Studies

For *IL1-RN* VNTR polymorphism, a total of 71 eligible studies met the preset inclusion criteria, in which 14,854 cases and 19,337 controls were included for the pooled analysis (Table S1). All studies were case–control studies, including 37 studies on gastric cancer, 6 on hepatocellular cancer, 4 on cervical cancer, 4 on breast cancer, 4 on lung cancer, and 16 on those categorized into other cancers. There were 40 studies of Asian descendents, 29 of Caucasian descendents and two with mixed ethnicity [15], [16]. Cancers were diagnosed histologically or pathologically in most studies. To analyze the VNTR in the second intron of *IL1-RN*, polymerase chain reaction (PCR) was performed in most studies, direct sequencing was used by two studies [17], [18], and the denaturing high performance liquid chromatography (DHPLC) was applied in one study [19]. In addition, most of the controls were sex- and age-matched for the case groups, of which 49 were population based and 22 were hospital based. In addition, 20 studies investigated the interactions between the polymorphism and *helicobacter pylori* (*H. pylori*), hepatitis B virus (HBV) or hepatitis C virus (HCV) infection status.

For *IL1B*+3954 polymorphism, 33 eligible studies contained 7,847 cases and 8,917 controls were enrolled for the pooled risk analysis (Table S2). Among the 33 studies, 14 studies discussed the risk of gastric cancer, 3 studies discussed the risk of oral cancer, 3 studies focused the risk of lung cancer, and 13 studies categorized in to other cancer. There were 14 studies of Asian descendents, 18 studies of Caucasian descendents, and one study with mixed ethnicity.

### Quantitative Synthesis

**Figure 1:**
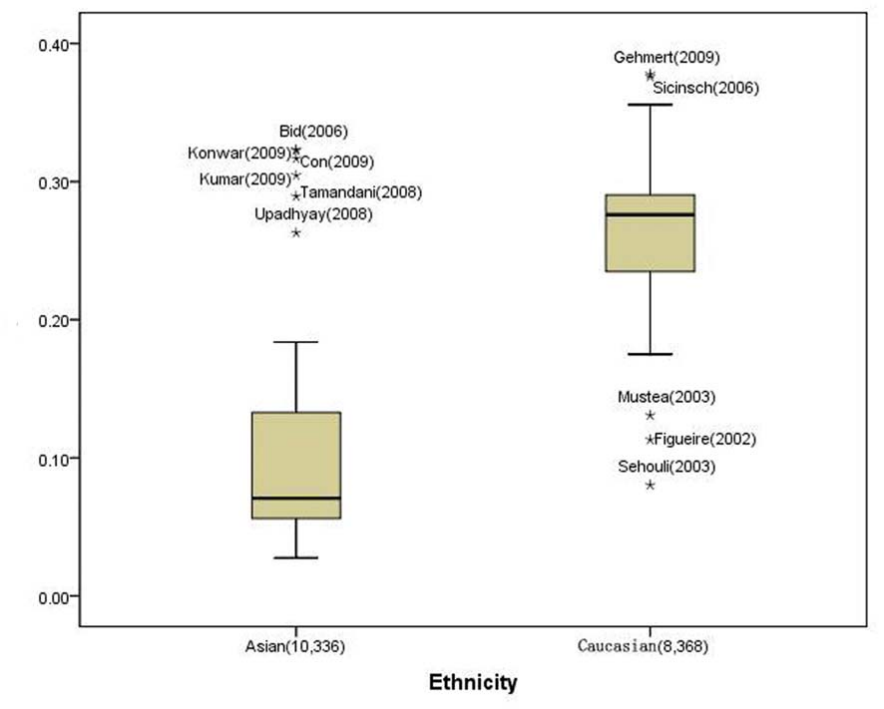
The allele frequency of the *IL1-RN* VNTR polymorphism in the controls may vary by ethnicity. Star or dot denotes outliers. The frequency of the 2 allele (Y axis) in Asian controls was lower than that in Caucasian controls (P = 0.000).

For *IL1-RN* VNTR polymorphism, a significant difference of *IL1-RN* -2 allele frequencies across different ethnicities was observed. The frequency of 2 allele was 11.14% (95% CI: 8.25–14.04) among Asian controls, which was significantly lower than that in Caucasian controls (26.05%; 95% CI: 23.41–28.69, *P*<0.001), as shown in Figure 1. Overall, a significant allelic association was observed in the whole study set (2 versus L: OR = 1.23, 95% CI: 1.10–1.38, Table 1). When grouped by cancer types, significant associations were still found in gastric cancer (2 versus L: OR = 1.20, 95% CI: 1.05–1.38, *P*_heterogeneity_<0.001), other cancer group (2 versus L: OR = 1.54, 95% CI: 1.12–2.13, *P*_heterogeneity_ = 0.000). In the genotypic analysis, significant associations were found in gastric cancer (2L versus LL: OR = 1.22, 95% CI: 1.05–1.41, *P*_heterogeneity_<0.001; 2L+22 versus LL: OR = 1.25, 95% CI: 1.09–1.43, *P*_heterogeneity_<0.001), other cancer group (2L versus LL: OR = 1.31, 95% CI: 1.00–1.72, *P*_heterogeneity_ = 0.000; 2L+22 versus LL: OR = 1.54, 95% CI: 1.12–2.11, *P*_heterogeneity_ = 0.000). In contrast to the increased risk of the *IL1-RN* polymorphism for cancers, a decreased risk was also observed in breast cancer (2L versus LL: OR = 0.74, 95% CI: 0.58–0.93, *P*_heterogeneity_ = 0.844; 2L+22 versus LL: OR = 0.78, 95% CI: 0.62–0.97, *P*_heterogeneity_ = 0.632). Significant increased risk was also observed in the ethnicities subgroup analysis. For Asian ethnicity, there was a significant association in the comparison of 2L versus LL (OR = 1.20, 95% CI: 1.01–1.42, *P*_heterogeneity_<0.001), 22+2L versus LL (OR = 1.25, 95% CI: 1.03–1.52, *P*_heterogeneity_<0.001) and allele 2 versus L (OR = 1.21, 95% CI: 1.00–1.47, Z = 1.96, *P* = 0.05, *P*_heterogeneity_<0.001). A significant association was also observed in the comparison of 22+2L versus LL (OR = 1.21, 95% CI: 1.07–1.36, *P*_heterogeneity_<0.001) and 2 versus L (OR = 1.22, 95% CI: 1.07–1.40, *P*_heterogeneity_<0.001) in Caucasian ethnicity. In addition, two studies with mixed ethnicity data revealed increased risk using the comparisons of 2L versus LL and 22+2L versus LL (2L versus LL: OR = 1.65, 95% CI: 1.17–2.33, *P*_heterogeneity_ = 0.096; 22+2L versus LL: OR = 1.57, 95% CI: 1.13–2.19, *P*_heterogeneity_ = 0.110). Subgroup analysis determined by the source of control revealed a significant association between the *IL1-RN* VNTR polymorphism and cancer risk, with the exception of hospital based controls for the comparison of 22 versus LL(2L versus LL: OR = 1.17, 95% CI: 1.04–1.32, *P*_heterogeneity_ = 0.000; 22+2L versus LL: OR = 1.21, 95% CI: 1.07–1.36, *P*_heterogeneity_ = 0.000; LL+2L versus 22: OR = 1.36, 95% CI: 1.01–1.84, *P*_heterogeneity_ = 0.000) and population based controls for the comparison LL+2L versus 22(22 versus LL: OR = 1.45, 95% CI: 1.06–1.41, *P*_heterogeneity_ = 0.000; 2L versus LL: OR = 1.19, 95% CI: 1.04–1.37, *P*_heterogeneity_ = 0.097; 22+2L versus LL: OR = 1.30, 95% CI: 1.12–1.51, *P*_heterogeneity_ = 0.000) summarized in Table 1.

**Table 1:** Stratification analyses of genetic susceptibility of *IL-1RN* VNTR polymorphism to cancer risk.

Category	n^a^	22 versus LL			2L versus LL			22+2L versus LL			LL+2L versus 22			2 versus L		
		OR(95%CI)	*P* b	*I* ^2^	OR(95% CI)	*P* b	*I* ^2^	OR (95% CI)	*P* b	*I* ^2^	OR(95% CI)	*P* b	*I* ^2^	OR(95% CI)	*P* b	*I* ^2^
Total	71	**1.37** **(1.07,1.75)** c	0.000	71.0	**1.19** **(1.07,1.32)** c	0.000	67.1	**1.25** **(1.12,1.41)** c	0.000	74.2	**1.27** **(1.00,1.61)** c	0.000	72.0	**1.23** **(1.10,1.38)** c	0.000	82.2
Cancer types																
Gastric cancer	37	1.20(0.85,1.69)^c^	0.000	65.6	**1.22** **(1.05,1.41)** c	0.000	61.0	**1.25** **(1.09,1.43)** c	0.000	60.4	1.09(0.77,1.54)^c^	0.000	71.3	**1.20** **(1.05,1.38)** c	0.000	73.3
Breast cancer	4	1.05(0.67,1.64)	0.554	0.0	**0.74** **(0.58,0.93)**	0.844	0.0	**0.78** **(0.62,0.97)**	0.632	0.0	1.14(0.74,1.76)	0.532	0.0	0.86(0.11,1.03)	0.276	22.4
Hepatocellular cancer	6	0.74(0.26,2.11)	0.710	0.0	1.16(0.89,1.51)	0.568	0.0	1.13(0.87,1.46)	0.449	0.0	0.73(0.26,2.10)	0.723	0.0	1.09(0.85,1.40)	0.353	9.9
Cervical cancer	4	**1.66** **(1.00,2.73)**	0.293	19.3	1.64(0.90,2.98)^c^	0.001	82.1	1.58(0.89,2.83)^c^	0.001	83.0	1.34(0.82,2.19)	0.559	0.0	1.40(0.90,2.19)^c^	0.002	79.8
Lung cancer	4	1.08(0.58,1.37)	0.155	42.8	0.92(0.81,1.03)^c^	0.095	52.9	0.92(0.74,1.14)^c^	0.038	64.4	1.09(0.87,1.38)	0.173	39.9	0.94(0.77,1.16)^c^	0.016	71.1
Other cancers	16	**2.06** **(1.16,3.65)** c	0.000	80.8	**1.31** **(1.00,1.72)** c	0.000	72.3	**1.54** **(1.12,2.11)** c	0.000	74.2	**1.89** **(1.10,3.26)** c	0.000	80.1	**1.54** **(1.12,2.13)** c	0.000	89.7
Ethnicities																
Asian	40	1.46(0.95,2.23)^c^	0.000	63.5	**1.20** **(1.01,1.42)** c	0.000	72.9	**1.25** **(1.03,1.52)** c	0.000	80.5	1.34(0.90,1.99)^c^	0.000	59.7	**1.21** **(1.00,1.47)** c	0.000	85.0
Caucasian	29	1.29(0.96,1.74)^c^	0.000	75.1	1.13(0.99,1.28)^c^	0.001	53.3	**1.21** **(1.07,1.36)** c	0.000	55.5	1.24(0.92,1.67)^c^	0.000	79.1	**1.22** **(1.07,1.00)** c	0.000	78.1
Mix	2	1.05(0.44,2.48)	0.924	0.0	**1.65** **(1.17,2.33)**	0.096	63.9	**1.57** **(1.13,2.19)**	0.110	60.8	0.87(0.37,2.02)	0.759	0.0	**1.36** **(1.17,1.27)**	0.292	82.2
Source of controls																
Population based	49	**1.45** **(1.06,1.99)** c	0.000	77.3	**1.19** **(1.04,1.37)**	0.097	29.5	**1.30** **(1.12,1.51)** c	0.000	80.6	1.02(0.82,1.27)	0.542	0.0	**1.29** **(1.11,1.49)** c	0.000	87.0
Hospital based	22	1.12(0.89,1.41)	0.695	0.0	**1.17** **(1.04,1.32)** c	0.000	73.9	**1.16** **(1.03,1.30)**	0.276	13.8	**1.36** **(1.01,1.84)** c	0.000	77.4	**1.11** **(1.01,1.22)**	0.534	0.00

a Number of studies.

b P value of Q-test for heterogeneity test.

c Random-effects model was used when a P value <0.05 for heterogeneity test; otherwise, fixed-effects model was used.

*I* ^2^: 0–25, no heterogeneity; 25–50, modest heterogeneity;.50 high heterogeneity.

For *IL1B*+3954 polymorphism, borderline significant risk associations were observed in the pooled analysis for the comparison of TT versus CC (OR = 1.17, 95% CI: 1.00–1.37, Z = 1.94, *P* = 0.053, *P*_heterogeneity = _0.155), TT+CT versus CC (OR = 1.15, 95% CI: 1.00–1.34, Z = 1.97, *P* = 0.049, *P*_heterogeneity_ = 0.000) and TT versus CT+CC(OR = 1.16, 95% CI: 1.00–1.35, Z = 1.99, *P* = 0.049, *P*_heterogeneity_ = 0.488) In addition, subgroup analysis revealed the similarly associations in the population based studies (TT versus CC: OR = 1.22, 95% CI: 1.00–1.49, Z = 1.95, *P* = 0.051, *P*_heterogeneity_ = 0.885; TT+CT versus CC:OR: 1.21; 95% CI:1.00–1.47, Z = 1.91, *P* = 0.056, *P*_heterogeneity_ = 0.000). Cancer type subgroup analysis revealed significant association for oral cancer (CT versus CC: OR = 0.65, 95% CI: 0.45–0.94; T/T+C/T versus CC: OR = 0.69, 95% CI: 0.49–0.98, *P* = 0.049), in contrast, significant association was observed in the other cancers subgroup(TT versus CC: OR = 1.51, 95% CI: 1.11–2.04; TT versus CT+CC: OR = 1.3, 95% CI: 1.06–1.75) summarized in Table 2.

### IL1-RN VNTR Polymorphism and H.pylori, HBV/HCV Infection Status

The genotype distribution of the *IL1-RN* VNTR genotype among cases and infection-matched controls was available in twenty studies that investigated gastric cancer infected by *H. pylori* and hepatocellular cancer infected by the hepatitis B or hepatitis C virus. Carriers of the 2 allele had higher cancer risk than those with the *IL1-RN-* L allele among gastric cancer studies, and the subgroup analysis results revealed that the L allele is a risk of gastric cancer independent of *H.pylori* infection. However, there was no association between the *IL1-RN* VNTR polymorphism and cancer risk in hepatocellular cancer studies as illustrated in Table 3.

### Test of Heterogeneity

For *IL1-RN* VNTR and *IL1B*+3954 polymorphism, there was significant heterogeneity across the studies. For this, the source of heterogeneity was explored for the heterozygote comparison (CT versus CC) by cancer type, ethnicity and source of controls. Cancer type (χ2 = 8.9, df = 3, *P* = 0.031), ethnicity (χ2 = 13.87, df = 2, P = 0.01) and source of controls (χ2 = 10.25, df = 2, *P = *0.006) contributed to the heterogeneity. In similarly, for *IL1-RN* VNTR polymorphism, Cancer type (χ2 = 52.17, df = 6, *P* = 0.000) but not ethnicity (χ2 = 5.63, df = 2, *P* = 0.060) or source of controls (χ2 = 0.42, df = 1, *P* = 0.518) contributed substantially to that heterogeneity.

Sensitivity analysis revealed that ten [20], [21], [22], [23], [24], [25], [26], [27], [28], [29] and four [30], [31], [32], [33] independent studies were the main cause of heterogeneity for *IL1-RN* VNTR and *IL1B*+3954, respectively. The heterogeneity was decreased when these studies were removed (*IL1-RN* VNTR 2L versus LL: *P*_heterogeneity = _0.065, *I* ^2^ = 22.6%; *IL1B*+3954 CT versus LL: *P*_heterogeneity = _0.097, *I* ^2^ = 26.4%).

### Publication Bias

**Figure 2:**
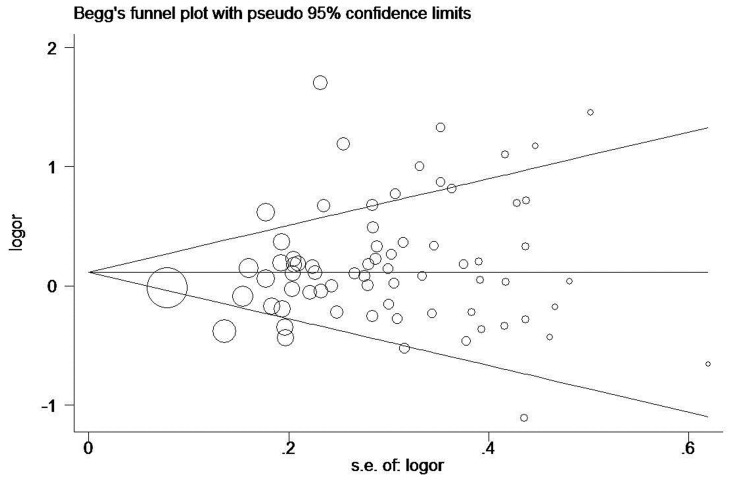
Begg’s funnel plot for publication bias test. 2L versus LL. Each circle denotes an independent study for the indicated association. Log[OR], natural logarithm of OR. Horizontal line stands for mean effect size.

**Figure 3:**
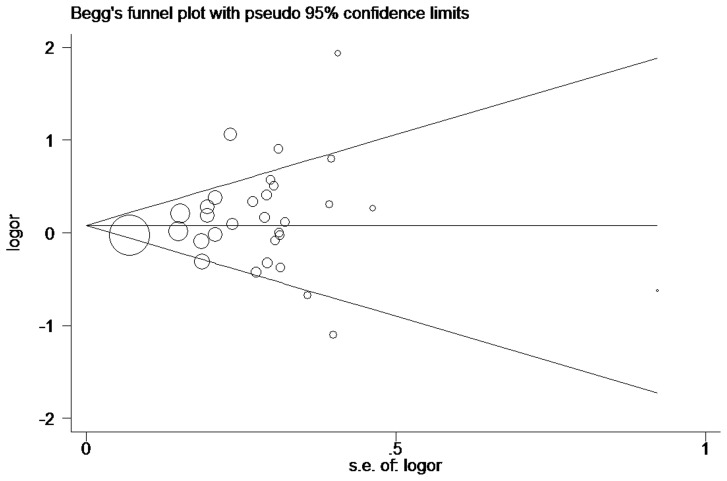
Begg’s funnel plot for publication bias test. CT versus CC. Each circle denotes an independent study for the indicated association. Log[OR], natural logarithm of OR. Horizontal line stands for mean effect size.

Begg’s funnel plot and Egger’s test were performed to assess the publication bias of the currently available literature. The shape of the funnel plots did not reveal any evidence for obvious asymmetry in all comparison models. Then, the Egger’s test was used to provide statistical evidence for funnel plot symmetry. The results still did not show any evidence of publication bias (*IL1-RN* VNTR: *t* = 1.84, *P* = 0.071 for 2L versus LL, Figure 2; *IL1B*+3954: *t* = 0.82, *P* = 0.420 for CT versus CC, Figure 3).

**Table 2:** Stratification analyses of genetic susceptibility of *IL1B+*3954 polymorphism to cancer risk.

Category	n^a^	TT versus CC			CT versus CC			TT+CT versus CC			TT versus CC+CT		
		OR(95% CI)	*P* b	*I* ^2^	OR(95% CI)	*P* b	*I* ^2^	OR (95% CI)	*P* b	*I* ^2^	OR(95% CI)	*P* b	*I* ^2^
Total	33	**1.17(1.00,1.37)**	0.155	20.9	1.13(0.98,1.31) c	0.000	65.9	**1.15(1.00,1.34)** c	0.000	69.1	**1.16(1.00,1.35)** c	0.488	0.0
Cancer types													
Gastric cancer	14	1.01(0.75,1.35)	0.276	17.6	**1.16(1.03,1.32)** c	0.001	62.8	1.24(0.99,1.54) c	0.000	65.7	0.99(0.74,1.32)	0.317	13.3
Oral cancer	3	0.86(0.47,1.55)	0.810	0.0	**0.65(0.45,0.94)**	0.978	0.0	**0.69(0.49,0.98)**	0.983	0.0	1.02(0.53,1.96)	0.858	0.0
Lung cancer	3	1.16(0.89,1.51)	0.155	0.0	1.05(0.93,1.18)	0.098	56.9	1.06(0.95,1.19)	0.081	60.2	1.14(0.88,1.48)	0.423	0.0
Other cancers	13	**1.51 (1.11,2.04)**	0.200	24.1	1.14(0.98,1.33) c	0.000	73.5	1.16(0.83,1.61) c	0.000	76.9	**1.36(1.06,1.75)**	0.540	0.0
Ethnicities													
Asian	14	1.19(0.78,1.82)	0.275	17.7	1.33(0.95,1.84) c	0.000	74.3	1.35(0.97,1.90) c	0.000	76.7	1.12(0.73,1.71) c	0.341	10.8
Caucasian	18	1.13(0.94,1.35)	0.163	24.8	1.00(0.87,1.15) c	0.032	42.0	1.02(0.89,1.18) c	0.011	48.8	1.14(0.97,1.35) c	0.516	0.0
Source of controls													
Population based	24	**1.22(1.00,1.49)**	0.885	0.0	1.17(0.96,1.42)^c^	0.000	71.2	**1.21(1.00,1.47)** c	0.000	73.2	1.20(0.98,1.46)	0.318	10.6
Hospital based	8	1.17(0.89,1.52)	0.072	32.4	1.04(0.92,1.17)	0.816	0.0	1.05 (0.94,1.18)	0.791	0.0	1.17(0.93,1.48)	0.894	0.0

a Number of studies.

b P value of Q-test for heterogeneity test.

c Random-effects model was used when a P value <0.05 for heterogeneity test; otherwise, fixed-effects model was used.

*I* ^2^ 0–25, no heterogeneity; 25–50, modest heterogeneity; 50 high heterogeneity.

**Table 3:** Bacterial/virus affection status Subgroup analysis of *IL1RN* VNTR polymorphism to cancer risk.

Category	22 versus LL			2L versus LL			22+2L versus LL			22 versus LL+2L			2 vs L		
	OR(95% CI)	*P* b	*I* ^2^	OR(95% CI)	*P* b	*I* ^2^	OR (95% CI)	*P* b	*I* ^2^	OR (95% CI)	*P* b	*I* ^2^	OR(95% CI)	*P* b	*I* ^2^
Total	**1.49** **(1.02,** **2.17)**	0.344	6.4	**1.41** **(1.22,** **1.64)**	0.070	29.9	**1.46** **(1.27,** **1.67)**	0.088	29.5	1.14(0.80,1.64)	0.497	0.0	**1.35** **(1.20,** **1.53)**	0.197	18.2
**Infection status**															
Positive-matched	1.33(0.86,2.04)	0.532	0.0	**1.36** **(1.07,** **1.74)**	0.015	46.2	**1.41** **(1.11,** **1.78)**	0.022	45.4	1.01(0.67,1.52)	0.553	0.0	**1.29** **(1.13,** **1.49)**	0.069	34.7
Negative-matched	2.17(0.98,4.78)	0.233	31.4	**1.64** **(1.19,** **2.25)**	0.895	0.0	**1.72** **(1.28,** **2.31)**	0.892	0.0	1.69(0.82,3.49)	0.344	6.4	**1.58** **(1.23,** **2.04)**	0.902	0.0
**Infection positive sugroup**															
*H.pylori*(gastric cancer)	1.51(0.93,2.43)	0.401	3.7	**1.55** **(1.12,** **2.15)**	0.020	49.1	**1.64** **(1.23,** **2.20)**	0.014	53.6	1.07(0.68,1.68)	0.335	12.2	**1.42** **(1.20,** **1.67)**	0.089	35.8
HBV/HCV (hepatocellular cancer)	0.74(0.26,2.11)	0.710	0.0	1.08(0.82,1.44)	0.616	0.0	1.06(0.80,1.67)	0.742	0.0	0.73(0.26,2.10)	0.723	0.0	1.03(0.79,1.33)	0.480	0.0

a Number of comparisons.

b P value of Q-test for heterogeneity test.

c Random-effects model was used when a P value <0.05 for heterogeneity test; otherwise, fixed-effects model was used.

## Discussion

In this meta-analysis, the VNTR polymorphism in intron 2 of *IL1-RN* was associated with increased risk for developing cancers of interest. Results from stratified analyses by cancer type indicated that the 2L genotype was associated with the increased risk of gastric cancer, and with increased cancer in Asian populations. Infection status analysis indicated that the 2 allele was a risk allele for gastric cancer independent of *H. pylori* status, but not hepatocellular cancer with HBV or HCV infection. For *IL1B*+3954 polymorphism borderline significant risk association was observed for cancer risk.

In the present study, this meta-analysis indicated a significant association of the *IL1-RN* VNTR polymorphism with gastric cancer, but not with breast cancer, hepatocellular cancers, cervical cancer or lung cancer. Our results suggested that the *IL1-RN* VNTR polymorphism may play different roles in the pathogenesis of different cancer types. Our findings are supported by evidence that the immune response is essential for gastric cancer development. It has been shown the 86 bp VNTR polymorphism of the *IL1-RN* has potential roles in regulating the immune response. The *IL1-RN*-2 allele had a high circulating IL1RA level and an even more elevated IL1β level [34]. Binding of IL1RA to the IL1 receptor inhibited IL1-mediated signaling, resulting in a strengthened and prolonged inflammatory response [35]. Moreover, El-Omar et al. found that the gastric cancer risk genotypes *IL1-RN*-2/2 were associated with decreased acid-secretion capacity [5]. Xue et al. reported *IL1-RN* -2 allele was associated with an increased risk of developing gastric carcinoma and even more significantly with noncardia gastric carcinoma or with intestinal-type gastric carcinoma [36], indicated *IL1-RN* VNTR polymorphism associated with different cancer risk according to the subtypes of gastric cancer and histological types. Furthermore, Wang et al. [37] has also revealed that *IL1-RN* -2 allele was associated with an increased risk of gastric cancer among Caucasians. Interestingly, in the cancer subtype analysis, we observed that the carriage of the 2 allele was found to act as a protective factor for breast cancer risk (Table 1). Our findings were not in accordance with some previous reports, which indicated inﬂammatory response might not be the major factor for the pathology of breast cancer [38], [39], [40]. However, other studies have suggested inflammatory response was closely related to breast cancer. It has been shown that IL1β may combine with the estrogen receptor-alpha, resulting in the enhancement of transcriptional activation in breast cancer cells [41]. Furthermore, it has been regarded that high levels of IL1β in peripheral blood is known to increase the level of leptin [42], which correlates with total body fat and stimulates the growth of mammary epithelium [43], [44], [45].

It is commonly known that *H. pylori* plays important roles in the pathology of gastric cancer [46]. Similarly, HCV and HBV are involved in the pathology of hepatocellular cancer [47]. It is important to perform the GxE analysis to evaluate the *IL1-RN* VNTR polymorphism effects for gastric cancer and hepatocellular cancer. Our results revealed that there was no interactions between the *IL1-RN* VNTR polymorphism and *H.pylori* infection in gastric cancer, suggesting that the risks for developing gastric carcinoma conferred by the *H.pylori* and *IL1-RN* VNTR genotypes may be independent. This may be due to different pathogenesis mechanisms underlying *H.pylori*-negative gastric cancer in contrast to *H.pylori*-positive gastric cancer [48]. The similar results were also reported by Wang et al. [37].

IL1β is well known to be a pro-inﬂammatory factor and mediate several immune responses in HCV infection [49]. Several independent studies provided evidence of associations between chronic hepatocellular cancer and chronic HCV or HBV infection [50]. However, our results from HBV/HCV infection matched studies that have shown negative interaction between the *IL1-RN* VNTR polymorphism and HBV/HCV infection in predisposition to hepatocellular cancers. More studies are needed to further evaluate the role of this polymorphism in virus-infected hepatocellular cancers.

*IL1B*+3954 polymorphism is a coding synonymous variant located in exon 5 of *IL1B*. The transition from cytosine to thymine does not change amino acid coding but may lead to an inactivation of the original splicing donor site. The alternative splicing results in a premature stop codon or exon skipping and produces a truncated protein that is likely to be rapidly degraded or functionally inactive [51]. *IL1B*+3954 polymorphism has been reported to influence the production of IL-1β protein [5], [51]. Meta-analysis results revealed *IL1B*+3954 had a borderline significant association with increased cancer risk. The pooled results were affected by individual studies, therefore, more studies should be cumulated to evaluate the role of the *IL1B*+3954 polymorphism in the etiology of cancer.

Although meta-analyses are robust, our study still has some limitations. First, most of the selected studies focused on gastric cancer. The relatively small sample size of studies in other stratified groups may lead to reduced statistical power. Second, although all eligible studies were summarized, the total sample size might have not been enough to make a convincing conclusion. When stratified analysis of tumor type, ethnicity or infection status was performed, the number of each subgroup was smaller. Third, our meta-analysis included no evaluation of potential gene-gene interactions and limited gene-environment interactions due to the lack of relevant published data. Because cancer is a complex disease, genetic factors may play a limited impact on the pathogenesis of cancers. In the results of our present meta-analysis, the *IL1-RN* VNTR had only moderate genetic susceptibility effects (ORs less than 2). More studies on large-scale gene-gene and gene-environment interactions are needed to further evaluate and elucidate the human genetic predisposition to various cancers.

In conclusion, this meta-analysis suggests that the *IL1-RN* VNTR polymorphism may contribute to genetic susceptibility to gastric cancer, which is closely related to the immunological response. *IL1B*+3954 has a borderline significant association with increased cancer risk. More studies are needed to further evaluate the role of the *IL1B*+3954 polymorphism in the etiology of cancer.

## Supporting Information

Figure S1 Process of study selection of case–control studies.
(TIF)

Table S1 Summary of published studies included for *IL1-RN* VNTR in present meta-analysis study.
(DOC)

Table S2 Summary of published studies included for *IL1B+3954* in present meta-analysis study.
(DOC)
