# Supplementary figures and images for: IL1 Receptor Antagonist Gene IL1-RN Variable Number of Tandem Repeats Polymorphism and Cancer Risk: A Literature Review and Meta-Analysis
Source: PLoS One. 2012 Sep 25;7(9):e46017. doi: 10.1371/journal.pone.0046017 (PMC3457944; doi:10.1371/journal.pone.0046017)

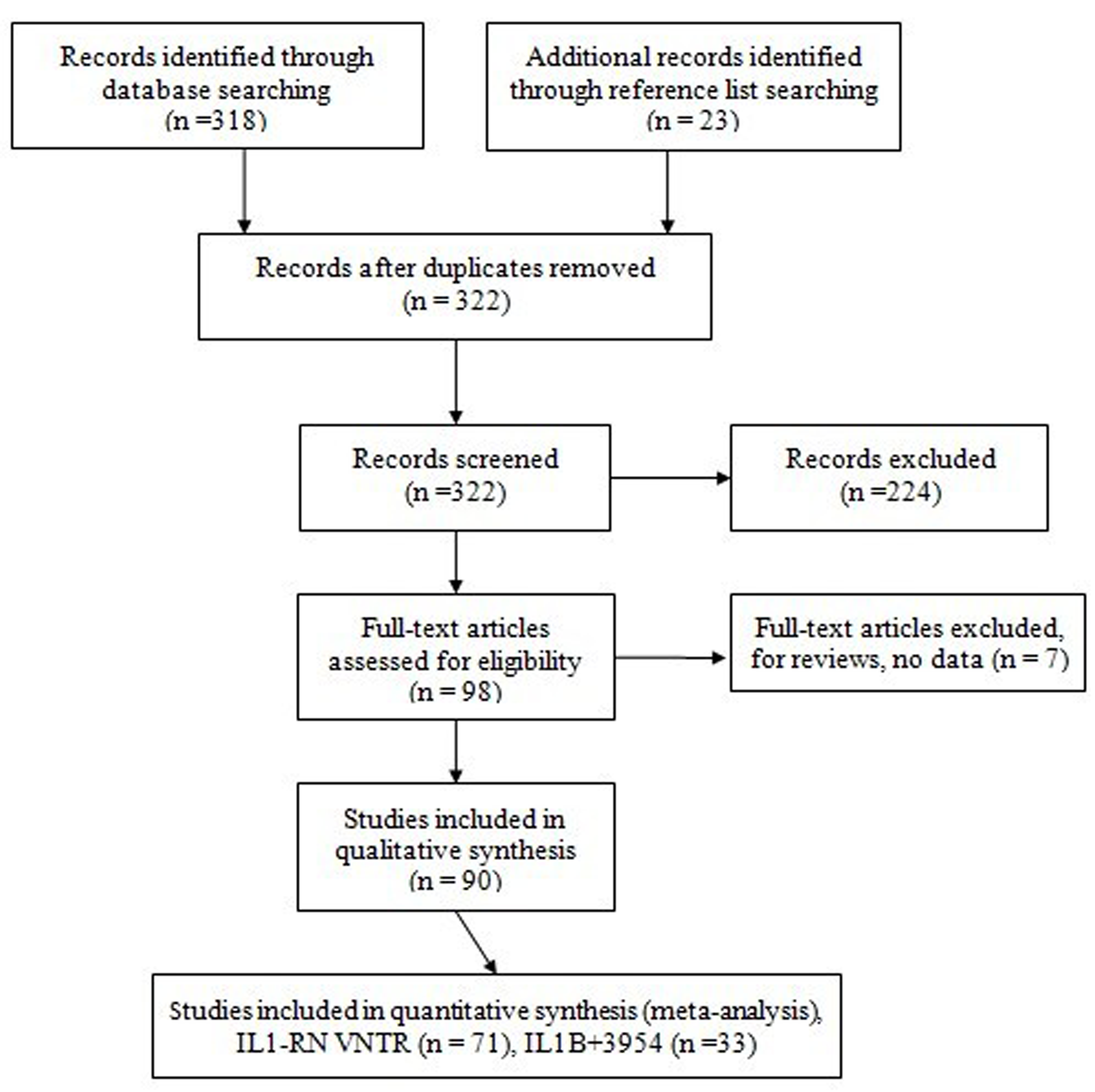

Supplement: Figure S1 — Process of study selection of case–control studies. (TIF) [file pone.0046017.s001.tif]
