# Supplementary material for: IL1 Receptor Antagonist Gene IL1-RN Variable Number of Tandem Repeats Polymorphism and Cancer Risk: A Literature Review and Meta-Analysis
Source: PLoS One. 2012 Sep 25;7(9):e46017. doi: 10.1371/journal.pone.0046017 (PMC3457944; doi:10.1371/journal.pone.0046017)
Supplement: Table S1 — Summary of published studies included for IL1-RN VNTR in present meta-analysis study. (DOC) [file pone.0046017.s002.doc]

**Supplement Table 1.** Summary of published studies included for *IL1-RN* VNTR in present meta-analysis study.

| First author | Publish year | Country | ethnicity | cancer type | Source | Ca(n) | Co(n) |
| --- | --- | --- | --- | --- | --- | --- | --- |
| Langabeer | 1998 | United kingdom | Caucasian | acute myeloid leukaemia | PB | 334 | 156 |
| Hulkkonen | 2000 | Finland | Caucasian | chronic lymphocytic leukemia | PB | 33 | 392 |
| Foster | 2000 | USA | Caucasian | Kaposi sarcoma | HB | 112 | 124 |
| Zheng | 2000 | Sweden | Caucasian | malignant lymphoma | PB | 72 | 129 |
| El-Omar | 2000 | USA | Caucasian | gastric cancer | PB | 366 | 429 |
| Matsuo | 2001 | Japan | Asian | malignant lymphoma | HB | 372 | 241 |
| Figueiredo | 2002 | Portugal | Eruopean | gastric cancer | PB | 222 | 221 |
| Ito | 2002 | Japan | Asian | breast cancer | HB | 227 | 185 |
| El-Omar | 2003 | USA | Caucasian | mix | PB | 475 | 216 |
| Mustba | 2003 | Germany | Caucasian | cervical cancer | HB | 113 | 107 |
| Wang | 2003 | Japan | Asian | hepatocellular cancer | HB | 125 | 149 |
| Lee | 2003 | Korean | Asian | gastric cancer | PB | 190 | 172 |
| Machado | 2003 | Portugal | Caucasian | gastric cancer | PB | 271 | 306 |
| Zeng | 2003 | China | Asian | gastric cancer | PB | 170 | 361 |
| Heneghan | 2003 | China | Asian | hepatocellular cancer | PB | 98 | 91 |
| Rollinson | 2002 | United kingdom | Caucasian | gastric marginal zone lymphoma | PB | 59 | 162 |
| Tanaka | 2003 | Japan | Asian | hepatocellular cancer | HB | 146 | 218 |
| Sehouli | 2003 | Germany | Caucasian | ovarian cancer | PB | 108 | 112 |
| Wu | 2003 | China | Asian | gastric cancer | PB | 220 | 230 |
| Hellmig | 2004 | Multi-country | Caucasian | gastric marginal zone lymphoma | HB | 151 | 343 |
| Hartland | 2004 | United kingdom | Caucasian | gastric cancer | PB | 57 | 264 |
| Wu | 2004 | China | Asian | MALT-lymphoma | PB | 75 | 321 |
| Glas | 2004 | Gemany | Caucasian | gastric cancer | PB | 88 | 145 |
| Gatti | 2004 | Brazil | Caucasian | gastric cancer | PB | 56 | 114 |
| Chen | 2004 | Taiwan | Asian | gastric cancer | PB | 142 | 164 |
| Lu | 2005 | China | Asian | gastric cancer | PB | 250 | 300 |
| Taguchi | 2005 | Japan | Asian | gastric cancer | PB | 365 | 238 |
| Zhang | 2005 | China | Asian | gastric cancer | PB | 154 | 166 |
| Zambon | 2005 | Italy | Caucasian | gastric cancer | PB | 129 | 654 |
| Vilaichone | 2005 | Thailand | Asian | gastric cancer | HB | 39 | 91 |
| Sakuma | 2005 | Japan | Asian | gastric cancer | PB | 140 | 103 |
| Perri | 2005 | Italy | Caucasian | gastric cancer | PB | 184 | 362 |
| Chen | 2005 | China | Asian | hepatocellular cancer | HB | 572 | 383 |
| Rocha | 2005 | Brazil | mix | gastric cancer | PB | 166 | 536 |
| Lind | 2005 | Norway | Caucasian | lung cancer | PB | 340 | 410 |
| Ruzzo | 2005 | Italy | Caucasian | gastric cancer | PB | 138 | 100 |
| Hefler | 2005 | Gemany | Caucasian | breast cancer | PB | 259 | 215 |
| Palli | 2005 | Italy | Caucasian | gastric cancer | PB | 185 | 537 |
| Chang | 2005 | Korea | Asian | gastric cancer | PB | 234 | 434 |
| Garza-Gonzalez | 2005 | Mexico | Caucasian | gastric cancer | HB | 25 | 201 |
| Sicinschi | 2006 | USA | Caucasian | gastric cancer | HB | 172 | 348 |
| Li | 2007 | China | Asian | gastric cancer | PB | 143 | 264 |
| Hirankarn | 2006 | Thailand | Asian | hepatocellular cancer | HB | 46 | 90 |
| Hu | 2006 | China | Asian | lung cancer | PB | 885 | 1024 |
| Bid | 2006 | India | Asian | bladder cancer | PB | 120 | 150 |
| Lee | 2006 | Korea | Asian | breast cancer | PB | 559 | 502 |
| Starzynska | 2006 | Poland | Caucasian | gastric cancer | HB | 112 | 109 |
| Kim | 2006 | Korea | Asian | gastric cancer | PB | 237 | 474 |
| Engels | 2007 | USA | Caucasian | lung cancer | PB | 1472 | 1626 |
| Zhang | 2007 | China | Asian | gastric cancer | HB | 214 | 230 |
| Ito | 2007 | Japan | Asian | mix | PB | 350 | 136 |
| Shin | 2008 | Korea | Asian | gastric cancer | PB | 122 | 100 |
| Singh | 2008 | Indian | Asian | cervical cancer | PB | 150 | 162 |
| Vishnoi | 2008 | Indian | Asian | gallbladder cancer | HB | 124 | 166 |
| Tamandani | 2008 | Indian | Asian | cervical cancer | PB | 140 | 209 |
| Upadhyay | 2008 | Indian | Asian | esophageal cancer | PB | 159 | 194 |
| Erzin | 2008 | Turkey | Caucasian | gastric cancer | HB | 33 | 60 |
| Ando | 2009 | Japan | Asian | gastric cancer | HB | 325 | 188 |
| Gehmert | 2009 | USA | Caucasian | gastric cancer | HB | 133 | 133 |
| Con | 2009 | Japan | Asian | gastric cancer | HB | 52 | 191 |
| Konwar | 2009 | Indian | Asian | breast cancer | HB | 100 | 200 |
| Al-Moundhri | 2009 | Oman | Asian | gastric cancer | PB | 107 | 107 |
| Ahirwar | 2009 | Indian | Asian | bladder cancer | PB | 213 | 287 |
| El-Din NK | 2009 | Egypt | Caucasian | Brain Tumors | PB | 15 | 97 |
| Kumar | 2009 | Indian | Asian | gastric cancer | PB | 136 | 110 |
| Persson | 2009 | Sweden | Caucasian | gastric cancer | PB | 65 | 296 |
| Barbosa | 2009 | Brazil | mix | gastric cancer | PB | 30 | 97 |
| Qian | 2010 | China | Asian | cervical cancer | PB | 404 | 404 |
| Okamoto | 2010 | Japan | Asian | hepatocellular cancer | HB | 88 | 83 |
| He | 2011 | China | Asian | gastric cancer | PB | 392 | 508 |
| Lim | 2011 | Singaporean | Asian | lung cancer | HB | 294 | 710 |

HB, hospital based; PB, population based; Ca, cases; Co, controls
