# Supplementary material for: IL1 Receptor Antagonist Gene IL1-RN Variable Number of Tandem Repeats Polymorphism and Cancer Risk: A Literature Review and Meta-Analysis
Source: PLoS One. 2012 Sep 25;7(9):e46017. doi: 10.1371/journal.pone.0046017 (PMC3457944; doi:10.1371/journal.pone.0046017)
Supplement: Table S2 — Summary of published studies included for IL1B+3954 in present meta-analysis study. (DOC) [file pone.0046017.s003.doc]

**Supplement Table 2.** Summary of published studies included for *IL1B +3954* in present meta-analysis study.

| First author | Publish year | Country | ethnicity | cancer type | Source | Ca(n) | Co(n) |
| --- | --- | --- | --- | --- | --- | --- | --- |
| Foster | 2000 | USA | Caucasian | kaposi sarcoma | HB | 111 | 122 |
| Zheng | 2000 | Sweden | Caucasian | mutiople myeloma | PB | 73 | 129 |
| Barber | 2000 | Scotland | Caucasian | pancreatic cancer | PB | 64 | 101 |
| El-Omar | 2000 | USA | Caucasian | gastric cancer | PB | 366 | 429 |
| Zeng | 2003 | China | Asian | gastric cancer | PB | 170 | 361 |
| Heneghan | 2003 | China | Asian | hepatocellular cancer | mixed | 98 | 267 |
| Hellmig | 2004 | Multi-country | Caucasian | mix | HB | 141 | 333 |
| Grimm | 2004 | Austria | Caucasian | vulvar cancer | PB | 67 | 228 |
| Hartland | 2004 | United kingdom | Caucasian | gastric cancer | PB | 59 | 286 |
| Glas | 2004 | Gemany | Caucasian | gastric cancer | PB | 88 | 145 |
| Zhang | 2005 | China | Asian | gastric cancer | PB | 154 | 166 |
| Tsai | 2005 | China | Asian | bladder cancer | PB | 130 | 105 |
| Chen | 2005 | China | Asian | hepatocellular cancer | HB | 573 | 385 |
| Hefler | 2005 | Gemany | Caucasian | breast cancer | PB | 269 | 227 |
| Palli | 2005 | Italy | Caucasian | gastric cancer | PB | 185 | 546 |
| Sakuma | 2005 | Japan | Asian | gastric cancer | PB | 140 | 103 |
| Tsai | 2005 | China | Asian | oral cancer | PB | 130 | 105 |
| Kaouther | 2005 | Tunisia | African | breast carcinoma | PB | 305 | 200 |
| Sicinschi | 2006 | USA | Caucasian | gastric cancer | HB | 137 | 262 |
| Bid | 2006 | India | Asian | bladder cancer | PB | 120 | 150 |
| Zhang | 2007 | China | Asian | gastric cancer | HB | 214 | 230 |
| Abazis-stamboulieh | 2007 | greece | Caucasian | mutiople myeloma | PB | 74 | 160 |
| Sobti | 2007 | India | Asian | cervical cancer | PB | 148 | 179 |
| Vairaktaris | 2007 | Greece | Caucasian | oral cancer | PB | 108 | 156 |
| Eleftherios | 2008 | Greece | Caucasian | oral cancer | PB | 108 | 156 |
| Ter-Minassian | 2008 | Canada | Caucasian | lung cancer | HB | 2150 | 1492 |
| Con | 2009 | Japan | Asian | gastric cancer | HB | 243 | 191 |
| Kumar | 2009 | Indian | Asian | gastric cancer | PB | 136 | 110 |
| Persson | 2009 | Sweden | Caucasian | gastric cancer | PB | 284 | 242 |
| Persson | 2009 | Sweden | Caucasian | gastric cancer | HB | 65 | 297 |
| Al-Moundhri | 2009 | Oman | Asian | gastric cancer | PB | 118 | 245 |
| Landvik | 2009 | Norway | Caucasian | lung cancer | PB | 357 | 430 |
| Kiyohara | 2010 | Japan | Asian | lung cancer | PB | 462 | 379 |

HB, hospital based; PB, population based; Ca, cases; Co, controls
